# Inflammatory and neuropathic pain animals exhibit distinct responses to innocuous thermal and motoric challenges

**DOI:** 10.1186/1744-8069-2-1

**Published:** 2006-01-05

**Authors:** Rami Jabakhanji, Jennifer M Foss, Hugo H Berra, Maria V Centeno, A Vania Apkarian, Dante R Chialvo

**Affiliations:** 1 Department of Physiology, Northwestern University Feinberg School of Medicine, 303 East Chicago Ave, Chicago IL, 60611, USA

## Abstract

Most current methods for assessing pain in animals are based on reflexive measures and require constant interaction between the observer and the animal. Here we explore two new fully automated methods to quantify the impact of pain on the overall behavior of the organism. Both methods take advantage of the animals' natural preference for a dark environment. We used a box divided into two compartments: dark and bright. In the motoric operant task, "AngleTrack", one end of the box was raised so that the animals had to climb uphill to go from the light to the dark compartment. In the thermal operant task, "ThermalTrack", the floor of the dark compartment was heated to a given temperature, while the light compartment remained at 25°C. Rats were individually placed in the light box and their crossing between chambers monitored automatically for 30 minutes. The angle of the box, or the temperature of the dark compartment, was altered to challenge the animals' natural preference. We test the hypothesis that different models of pain (inflammatory or neuropathic) can be differentiated based on performance on these devices. Three groups of rats were tested at five different challenge levels on both tasks: 1) normal, 2) neuropathic injury pain (Spared Nerve Injury), and 3) inflammatory pain (intraplantar injection of Carrageenan). We monitored the position of the animals as well as their rate of switching between compartments. We find significant differences between the three groups and between the challenge levels both in their average position with respect to time, and in their switching rates. This suggests that the angle-track and thermal-track may be useful in assessing automatically the global impact of different types of pain on behavior.

## Introduction

Assessing the level of pain in animals is a key element in pain research. Current pain assessment techniques are mainly limited to estimating animal responses elicited by stimuli to the, presumably, affected area using either: 1) thermal (paw immersion test, hot plate test, radiant heat test, acetone test), 2) tactile (Von Frey filaments), and/or 3) mechanical (paw pressure) stimuli. These responses are invariably reflexive in nature, and are informative only about the thresholds of the sensory variables being probed, thermal, tactile, or mechanical. These thresholds are obviously important in the assessment of painful states, but are not the complete perceptual pain experience from a whole organism perspective. Assessing pain perception using reflexes is also problematic, because responses are present even in anesthetized, spinalized, and de-cerebrate animals, all questionable conditions, for more extensive discussion of these issues see [1,2]. Finally, an important methodological concern is the variability of most current techniques, which involves a significant level of animal-experimenter interaction, making differences in the test execution and in the manipulation of animals between experimenters confounding factors [3].

A long list of human brain imaging studies has repeatedly shown the involvement of the cortex in human pain perception [4]. Consistent and guided by these results multiple groups have begun charting the contribution of the cortex to pain behavior in the rat, and demonstrated modulation of spinal cord nociceptive neurons with cortical manipulations [5-9]. Consequently, more objective methods are desirable to assess global pain behavior in unrestrained, awake, behaving animals, which incorporate the contribution of the cortex in pain conditions. Recently, we reported a new automated method to assess learned responses to acute thermal pain in rats [1]. As expected, the measured responses collected with this method, differed significantly between healthy and neuropathic rats, and between types of neuropathic rats. An important drawback of this method, however, was its learning dependent nature. Thus animals often did not give consistent results until after a few training sessions.

In this paper we introduce two variants, motoric and thermal, of a novel and fully automated pain assessment technique based on an operant conditioning task able to provide meaningful data of the impact of pain conditions on place preference in a single session and with no training requirements. We test these methods in a neuropathic and inflammatory pain conditions, and show differential modulation of behavior in each type of pain condition.

## Results

**Figure 1:**
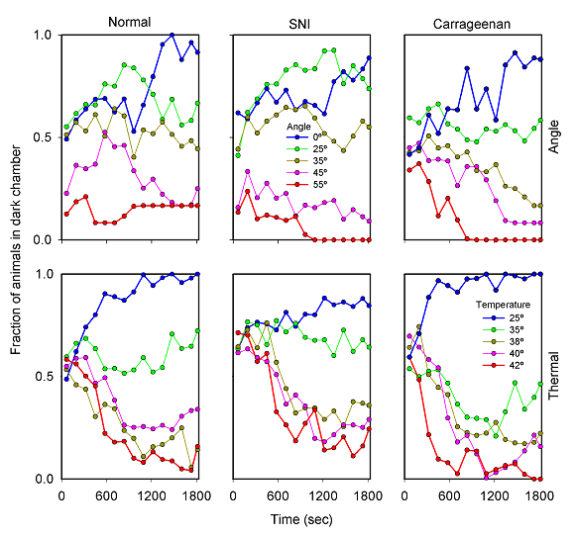
Average animal behavior as a function of time for the three groups on both operant tasks. In all cases the fraction of animals present in the dark chamber is plotted as a function of time. Symbol colors are used to indicate (from blue to red) progressive increases in challenge, for both tasks (indicated in the insets, as inclination angle or dark chamber surface temperature).

**Figure 2:**
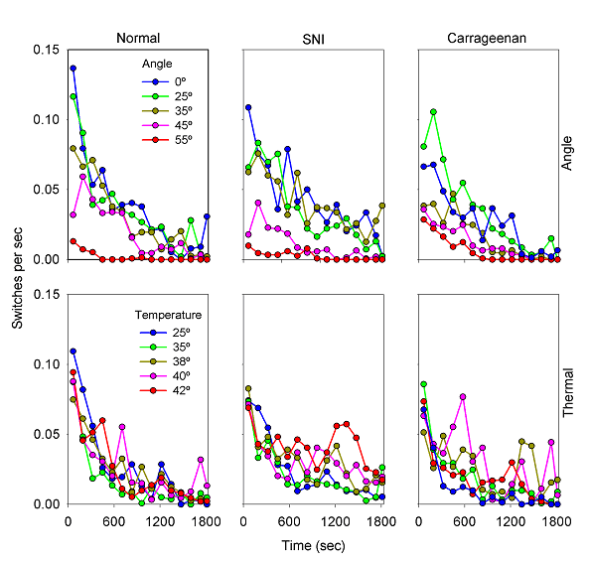
Estimation of the average mobility. Average number of movements from one chamber to the other (switches), per second, per animal, as a function of challenge level and time. Legends are similar to figure 1.

**Figure 3:**
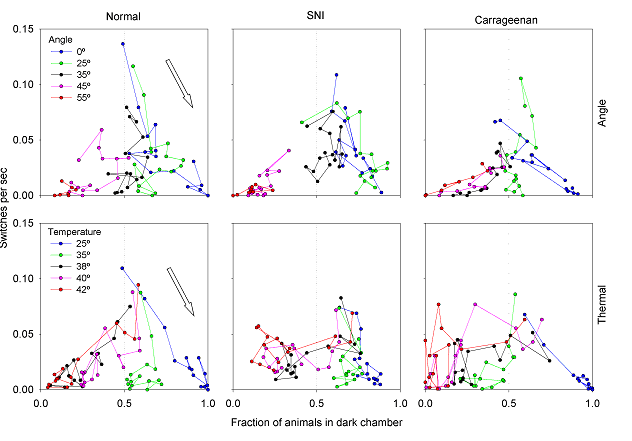
Composite plot of the data presented in figures 1 and 2. Fraction of animals in the dark is plotted on the x-axis as a function of the average mobility on the y-axis. Color code is the same as in figures 1 and 2. The plot demonstrates the inter-relationship between location and mobility for different challenges and different groups of rats. The arrows illustrate the typical time evolution, for one condition. Behavior usually begins at high mobility and spending equal times in both chambers and ends with low mobility and staying in the preferred chamber. This normal pattern is disrupted to different extents in the injured animals.

**Figure 4:**
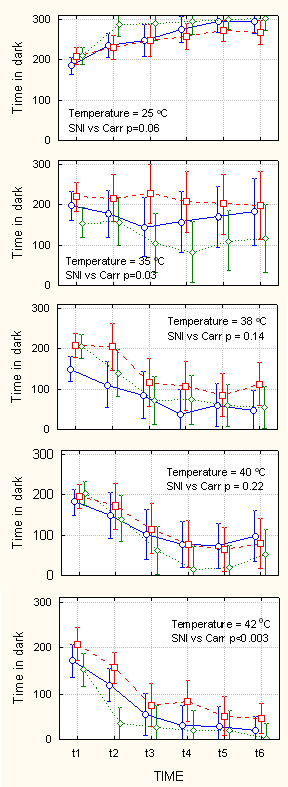
Behavior on ThermalTrack as a function of increasing levels of challenge. Amount of time spent in the dark compartment in seconds (maximum = 300 seconds), as a function of floor temperature in the dark compartment (indicated in each panel ranging from 25–42°C), while light compartment floor temperature is kept at 25°C, over 30 minutes of monitoring (t1 – t6 are 5-minute consecutive windows), and as a function of type of injury (blue circles = control, red squares = SNI, green diamonds = Carrageenan rats). In each panel, statistical outcome of planned comparison between SNI and Carrageenan (Carr) groups are indicated. There is no difference between SNI and Carr groups at intermediate levels of challenge. However, the two groups of animals behave differently at low and high levels of challenge. Bars are 95% confidence intervals. Note that variability increases for intermediate challenge levels.

**Figure 5:**
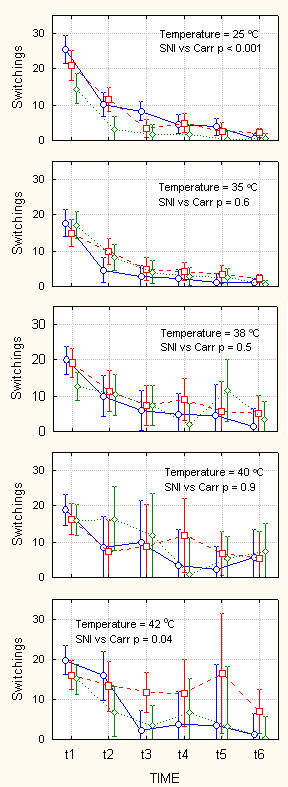
Mobility on ThermalTrack as a function of increasing levels of challenge. Number of switches from one compartment to the other (within 300 seconds time windows), as a function of floor temperature in the dark compartment (indicated in each panel, ranging from 25–42°C), while light compartment floor temperature is kept at 25°C, over 30 minutes of monitoring (t1 – t6 are 5-minute consecutive windows), and as a function of type of injury (blue circles = control, red squares = SNI, green diamonds = Carrageenan rats). In each panel, statistical outcome of planned comparison between SNI and Carrageenan (Carr) groups are indicated. There is no difference between SNI and Carr groups at intermediate levels of challenge. However, the two groups of animals behave differently at low and high levels of challenge. Bars are 95% confidence intervals. Note the increase in variability of behavior with increase in challenge level.

**Figure 6:**
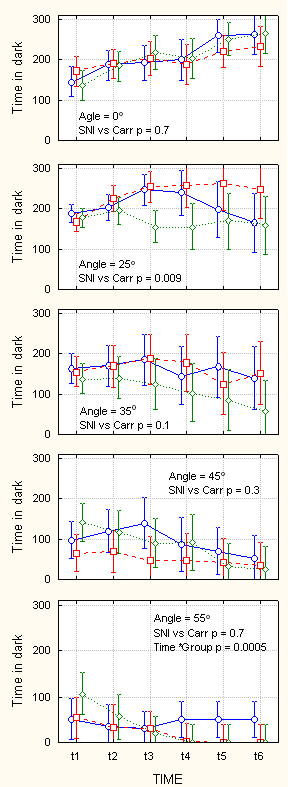
Behavior on Angle as a function of increasing levels of challenge. Amount of time spent in the dark compartment in seconds (maximum = 300 seconds), as a function of inclination of the dark compartment (indicated in each panel ranging from 0–55°), while light compartment floor is kept at 0°, over 30 minutes of monitoring (t1 – t6 are 5-minute consecutive windows), and as a function of type of injury (blue circles = control, red squares = SNI, green diamonds = Carrageenan rats). In each panel, statistical outcome of planned comparison between SNI and Carrageenan (Carr) groups are indicated. The largest difference between SNI and Carr groups is at 25° inclination. At 55° inclination, there is a strong interaction between time and groups, where we observe no difference between groups for early time windows, and a large difference between control and injured animals. Bars are 95% confidence intervals. Note that variability increases for intermediate challenge levels in the later time bins.

**Figure 7:**
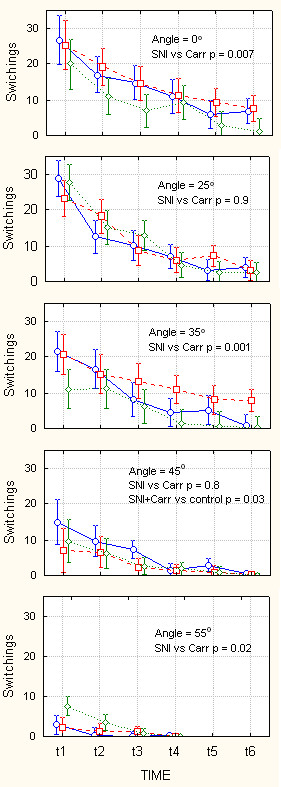
Mobility on AngleTrack as a function of increasing levels of challenge. Number of switches from one compartment to the other (within 300 seconds time windows), as a function of the dark compartment floor inclination (indicated in each panel, ranging from 0–55°), while light compartment floor is kept at 0°, over 30 minutes of monitoring (t1 – t6 are 5-minute consecutive windows), and as a function of type of injury (blue circles = control, red squares = SNI, green diamonds = Carrageenan rats). In each panel, statistical outcome of planned comparison between SNI and Carrageenan (Carr) groups are indicated. The largest difference between SNI and Carr groups are observed at 0° and 35° angle inclinations. Bars are 95% confidence intervals. Variability of behavior decreases mainly at highest challenge level.

**Figure 8:**
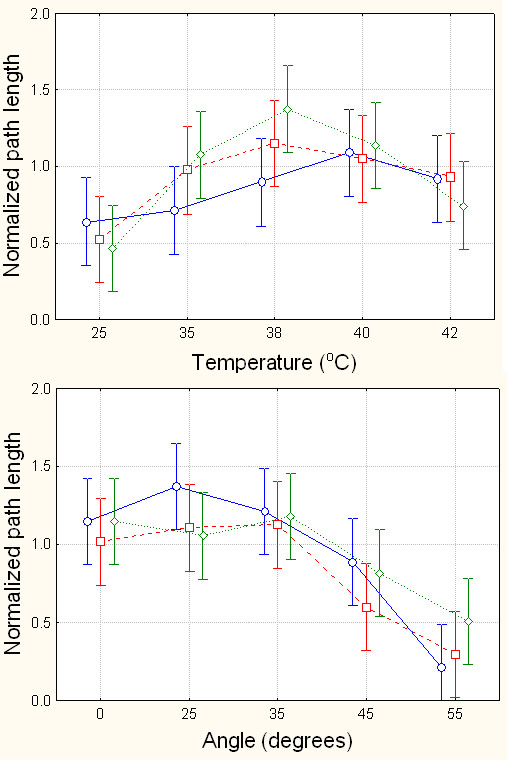
Path length as a function of challenge level for ThermalTrack (top) and AngleTrack (bottom), as a function of type of injury (blue circles = control, red squares = SNI, green diamonds = Carrageenan rats). Bars are 95% confidence intervals.

Modification of place preference by innocuous heat, ThermalTrack, and by a motoric challenge, AngleTrack, was assessed in 12 normal rats, 12 spared nerve injury (SNI) rats, and 12 Carrageenan injection induced inflammation rats. The outcomes, time spent in dark chamber and rate of switching between chambers, were analyzed across the three groups as a population, figures 1, 2, 3, and analyzed statistically based on individual animal responses, Tables I, II, III, IV and figures 4, 5, 6, 7, 8.

**Table 1:** Analysis of variance for ThermalTrack for time spent in black compartment

***Repeated Measures Analysis of Variance***					
***Effective hypothesis decomposition***					
***Effect***	***SS***	***Degr. of Freedom***	***MS***	***F***	***p***
***Intercept***	22002914	1	22002914	825.4245	0
***Group***	271754	2	135877	5.0973	0.007111
***Temp***	4818093	4	1204523	45.1869	0
Group*Temp	372302	8	46538	1.7458	0.091419
***Error***	4398320	165	26656		
***TIME***	775688	5	155138	41.7137	0
TIME*Group	36853	10	3685	0.9909	0.449507
TIME*Temp	1112005	20	55600	14.9499	0
TIME*Group*Temp	132656	40	3316	0.8917	0.663403
***Error***	3068262	825	3719		

Group: Control, SNI, Carrageenan;

Temperature = Temp: 25, 35, 38, 40, 42°C;

Time = 6, 5-minute intervals in 30 minutes.

**Table 2:** Analysis of variance for ThermalTrack for switches between compartments

***Repeated Measures Analysis of Variance***					
***Effective hypothesis decomposition***					
***Effect***	***SS***	***Degr. of Freedom***	***MS***	***F***	***p***
***Intercept***	64914.01	1	64914.01	303.5580	0.000000
***Group***	1196.87	2	598.44	2.7985	0.063797
***Temp***	1708.43	4	427.11	1.9973	0.097280
Group*Temp	1915.29	8	239.41	1.1196	0.352612
***Error***	35284.24	165	213.84		
***TIME***	25828.73	5	5165.75	56.5214	0.000000
TIME*Group	1437.34	10	143.73	1.5727	0.109973
TIME*Temp	1905.64	20	95.28	1.0425	0.408081
TIME*Group*Temp	3543.95	40	88.60	0.9694	0.526097
***Error***	75400.51	825	91.39		

Group: Control, SNI, Carrageenan;

Temperature = Temp: 25, 35, 38, 42°;

Time = 6 5-minute intervals in 30 minutes.

**Table 3:** Analysis of variance for AngleTrack for time spent in black compartment

***Repeated Measures Analysis of Variance***					
***Effective hypothesis decomposition***					
***Effect***	***SS***	***Degr. of Freedom***	***MS***	***F***	***p***
***Intercept***	18926051	1	18926051	662.8197	0.000000
***Group***	97940	2	48970	1.7150	0.183153
***Angle***	5164217	4	1291054	45.2147	0.000000
Group*Angle	301539	8	37692	1.3200	0.236748
***Error***	4711384	165	28554		
***TIME***	43302	5	8660	2.2578	0.046952
TIME*Group	86838	10	8684	2.2639	0.013004
TIME*Angle	543750	20	27187	7.0879	0.000000
TIME*Group*Angle	216322	40	5408	1.4099	0.049649
***Error***	3164485	825	3836		

Group: Control, SNI, Carrageenan;

Angle: 0°, 25°, 35°, 45°, 55°;

Time = 6, 5-minute intervals in 30 minutes.

**Table 4:** Analysis of variance for AngleTrack for switches between compartments

***Repeated Measures Analysis of Variance***					
***Effective hypothesis decomposition***					
***Effect***	***SS***	***Degr. of Freedom***	***MS***	***F***	***P***
***Intercept***	61849.93	1	61849.93	625.4035	0.000000
***Group***	1261.02	2	630.51	6.3755	0.002152
***angle***	19227.54	4	4806.89	48.6054	0.000000
Group*angle	2839.15	8	354.89	3.5886	0.000724
***Error***	16317.85	165	98.90		
***TIME***	26293.47	5	5258.69	165.5387	0.000000
TIME*Group	525.29	10	52.53	1.6536	0.087425
TIME*angle	5895.36	20	294.77	9.2790	0.000000
TIME*Group*angle	1555.48	40	38.89	1.2241	0.163869
***Error***	26207.90	825	31.77		

Group: Control, SNI, Carrageenan;

Angle: 0°, 25°, 35°, 45°, 55°;

Time = 6, 5-minute intervals in 30 minutes.

### Populational analysis of behavior

The animals' preference of one chamber over the other is measured by amount of time they spent in the respective compartment. Figure 1 shows the group overall time spent in the dark chamber. For both boxes, animals initially spend about 50% time in the two compartments, exploring their environment, and in time settle down and spend most of the time in the preferred compartment. Generally, figure 1 shows that the higher the challenge the less time the animals spend in the dark chamber (and more in the bright chamber), although we observe differences in the rate of change of this decision in time and in its starting point, between the three groups of animals. The data shows that at low levels of challenge the different animal groups behave differently. However, as the challenge level increases their behaviors become more similar and all three groups avoid the dark chamber at about the same rate.

The switches performed as a function of time measure mobility between compartments. Figure 2 shows switches per second, for all challenges studied for the three populations of rats. The animals begin the experiment with the most exploratory behavior, which is demonstrated by a higher mobility. As time passes, animals in all groups move less frequently. Moreover, the rats show less mobility for higher challenge levels, especially for the motoric task. In figure 3, we have plotted mobility and position against one another. This figure shows that in general, as the animals stop exploring, they settle into a position that is a function of the challenge level. This is mostly true for the healthy rats. The injured rats, however, show various abnormal patterns in comparison to the normal animals.

### Individual rat based analysis of behavior

The overall statistical evaluation for time spent in the dark chamber is shown for ThermalTrack in Table I, and for AngleTrack in Table III. These are 3-way repeated measures (in time and animals) ANOVA tests for amount of time spent in the dark chamber, in six consecutive 5-minute time-windows, as a function of animals, groups, and challenge levels. Group or Group*Time interaction are highly significant, indicating that both devices can distinguish between normal, SNI, and Carrageenan groups.

Overall statistical evaluation for mobility (switches between compartments in 6 consecutive 5-minute time-windows) is shown for ThermalTrack in table II and for AngleTrack in Table IV. On the ThermalTrack only Time is a significant factors, while Group is borderline significant (p < 0.07), and Time*Group interaction is also borderline significant. On the AngleTrack, Group, Angle, and Time are significant factors, and Group*Angle and Time *Angle interactions are also significant.

Figures 4, 5, 6, 7 show behavioral outcomes as measured in time spent in dark chamber (figures 4 and 6) and as measured in number of switches between chambers (figures 5 and 7), for the three groups of animals, as a function of challenge level, and time. In each set of panels and for each challenge level, outcome comparison is shown for planned comparison test between the two groups of injured animals (SNI vs. Carr, p-values). Figure 4 top panel shows duration of time spent in the dark chamber when the floor of both dark and bright chambers is at 25°C. All three groups of animals begin by spending 2/3 of time in the dark chamber and in time proceed to spend more time in this chamber, in the 6^th ^time-window they are in the dark chamber for > 90% of the time. Unexpectedly, in this case where there is no difference in floor temperature between the two chambers, there is a borderline difference between SNI treated and Carrageenan treated animals, with Carrageenan treated animals spending more time in the dark chamber. The difference in preference is consistent with mobility for the same groups at the corresponding challenge: Top panel of figure 5 shows evolution of mobility in time when floor temperature is 25°C in both compartments. The three groups of animals move between compartments between 15–25 times in the first 5 minutes. This value is reduced to 1–2 switches in the 6^th ^5-minute window. Moreover, there is a large difference between SNI treated and Carrageenan treated groups, with Carrageenan treated animals showing decreased mobility throughout the 30 minutes. Thus, in this baseline condition the Carrageenan group showed higher preference for the dark coupled with decreased mobility in contrast to the SNI group. At the next temperature challenge, 35°C for the floor of dark chamber vs. 25°C for the floor of the bright chamber (figure 4 2^nd ^panel), we observe a reversal of behavior. Now, the Carrageenan group shifts preference in time moving away from the dark and staying longer in the white chamber. In contrast the SNI group continues to spend 2/3 of the time in the dark chamber. The difference in duration spent in black chamber between SNI and Carrageenan groups is significant (p = 0.03), and the control animals behavior is intermediate to the two treated groups. At this challenge level, the difference in preference between SNI and Carrageenan groups is independent of mobility, since there are no differences between the two groups in switching events (figure 5, 2^nd ^panel). The next two challenge levels, 38°C and 40°C for the dark floor (figures 4 and 5, 3^rd ^and 4^th ^panels), there are no differences between SNI and Carrageenan groups both in time spent in dark chamber and in mobility. However, note that the general pattern seen for 35°C is preserved, i.e. the Carrageenan group tends to spend more time in the white chamber than the SNI group. This difference becomes more pronounced and statistically significant at the highest heat challenge tested, 42°C (figure 4, 5^th ^panel, p < 0.003), and at this level there is also a significant difference in mobility with Carrageenan group switching less often between chambers (figure 5, 5^th ^panel, p = 0.04). It is noteworthy that the variance of the behavior also shows characteristic changes: For time spent in the dark, variance (as expressed by 95% confidence intervals in figure 4) increases from the lowest challenge level (25°C) to intermediate challenge levels, and then decreases again especially at the highest challenge level (42°C). In contrast, for mobility, variance (as expressed by 95% confidence interval in figure 5) increases monotonically with increasing challenge levels.

Performance on AngleTrack is shown in Figure 6 and 7. Figure 6 top panel shows duration of time spent in the dark chamber when the floor of both dark and bright chambers have no inclination, Angle = 0°. All three groups of animals begin by spending 1/2 of time in the dark chamber and in time proceed to spend more time in this chamber, in the 6^th ^time-window they are in the dark chamber for about 250 seconds (of a maximum possible 300 seconds). There is no preference difference between the three groups. On the other hand, there is a large difference between Carrageenan and SNI in mobility for this baseline challenge (Figure 7, top panel, p = 0.007), which is similar to the difference seen in mobility for baseline ThermalTrack challenge. When the dark chamber is inclined at 25°, the three groups of animals show different pattern of preference shift. The SNI animals in time shift to spending more time in the dark pattern; while the Carrageenan animals shift away from the dark and tend to spend more time in the bright chamber. This difference is statistically significant (figure 6, 2^nd ^panel, p = 0.009). At this challenge level, there is no difference in mobility between groups (figure 7, 2^nd ^panel). For larger inclines 35°, 45°, and 55°, there is no difference between SNI and Carrageenan groups regarding time spent in the dark. However, at 55° incline, there is a significant time*Group interaction (p = 0.0005) due to the fact that normal animals continue to stay in the dark for about 50 seconds (in every 300 second time-window), while injured animals do not after 15 minutes from the start of monitoring (figure 6, 5^th ^panel). Despite of the lack of preference differences for these higher inclines, there are differences in mobility between SNI and Carrageenan groups at 35° incline and at 55° incline (figure 7, last three panels).

Normalized path length was calculated for ThermalTrack and AngleTrack, for all animal groups (figure 8). This is an integrated value, incorporating time spent in the dark and switching events between compartments, as an overall energy metric that indicates the change in behavior over the 30 minutes of monitoring. Since the measure is normalized, the outcome can be useful to compare performance between tasks. In figure 8 we observe that path length is larger for AngleTrack vs. ThermalTrack for low levels of challenge, and this pattern reverses for high levels of challenge. 2-way ANOVA for normalized path length shows only dependence on challenge level and not on animal groupings for both tracks: For ThermalTrack, temperature has p < 10^-5^, and group, and group*temperature are not significant; for AngleTrack, angle has p < 10^-6^, and group, and group*angle interaction are not significant. Despite lack of statistical differences, on the ThermalTrack we observe a systematic change in path length between the three animal groups, with the inverted u-shaped curve becoming sharper from control animals to SNI animals and to Carrageenan animals.

### Relationship between mechanical thresholds and operant tasks

**Figure 9:**
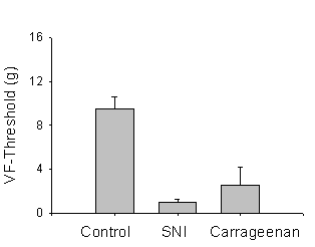
Mechanical sensitivity as measured with Von Frey thresholds (in grams) for the three groups of rats for the left hind paw. Mean and 95% confidence intervals are shown, n = 12 per group.

Tactile allodynia as assessed by determining Von Frey withdrawal thresholds was robust in both SNI and Carrageenan rats (Figure 9). We sought to test the relationship between this mechanical sensitivity and performance on the ThermalTrack and AngleTrack by correlating mechanical thresholds to position and switches, across the different challenge levels. In general, out of the many possible combinations, few weakly significant correlations were found between the two methods, mainly for ThermalTrack and only for a few specific temperatures. We do not report these correlations since they do not seem robust.

## Discussion

The presented results suggest that the AngleTrack and ThermalTrack may be useful in assessing the global impact of different types of pain on behavior. The main contribution of this work is the fact that we can use simple and objective behavioral measures (time spent in one chamber, switches between chambers) with which we can differentiate types of pain conditions from normal animals, and between the types of pain. The presented evidence suggests that the behavioral differences illuminated by these tests yield information not given by merely assessing tactile sensitivity in the traditional manner. Both inflammatory and neuropathic pain reduce the tactile threshold by about 80–90%, yet in many of the AngleTrack and ThermalTrack results, these injuries produce opposite changes in behavior. Furthermore, individual rat pain thresholds for the injured limb do not correlate with the animal's behavior on the AngleTrack or ThermalTrack tests.

The choice of the animal models requires some comments: We sought to test the hypothesis that neuropathic and inflammatory pain conditions differentially impact motoric and/or thermal challenges to the animals' natural preference of staying in the dark. For this purpose we chose to examine SNI and Carrageenan injured animals, contrasted between them and to control animals that are normal and are matched for age or body weight. We avoided the use of sham-operated animals as controls since these animals would then become controls for each respective injury, and moreover they would be contaminated by the sham operation induced inflammatory consequences. The SNI is our preferred choice model for neuropathy induced pain-like behavior since the surgical procedure is simple, causing less and consistent inflammatory damage, and since the behavioral outcome is highly consistent between animals. The Carrageenan injury results in obvious inflammation of the paw immediately after the injection. However, we examine behavior in these animals 12 hours later when the paw inflammation is subsided enough that it is not obvious to visual inspection and yet the animals continue to exhibit increased tactile sensitivity.

The 3-way ANOVA statistical analyses (Tables I, II, III, IV) indicate the effects of grouping of animals, time, and challenge level, and their interactions on the behavioral outcomes. Time is a main factor that significantly affects the outcomes for both tasks and for time spent in dark chamber as well as for switching events (p < 10^-6 ^in all 4 cases). This is a reflection of the presence of two behaviors: an initial exploratory phase characterized with high mobility and with spending approximately equal time in both chambers; followed by exhibition of a decision of preference where mobility is reduced and the animal spends more time in a specific chamber. As figures 1 and 2 illustrate, both time spent in the dark chamber (position) and mobility (switches) become stabilized by about 1000 seconds after start of task for ThermalTrack and AngleTrack. This is also reflected in figures 4, 5, 6, 7. Thus, future studies can be done in this smaller time window. On the other hand, figures 4, 5, 6, 7 also illustrate that there are group differences in outcomes for different challenge levels, at the initial exploratory phase (10 of 20 illustrated conditions in the 1^st ^5-minute time-window), at intermediate times where the animals have not settled to a final decision (10 of 20 cases in 3^rd ^and 4^th ^time-windows), and at later times where decision is more obvious (6 of 20 cases in 6^th ^time-window). Besides the effect of time, the 3-way ANOVA shows distinct outcomes regarding group, group*challenge, and group*challenge*time interactions for the two tests and for the two outcome measures, suggesting that these tests interact with the groups differentially.

The outcomes of the 2-way ANOVA for each test and outcome, at each challenge level are illustrated in figures 4, 5, 6, 7. On the ThermalTrack, performance at 35°C is most interesting since the three groups show distinct preference in the absence of mobility differences between them, and the preference of control animals is intermediate between SNI and Carrageenan groups. Similarly on the AngleTrack, performance at 25° inclination is distinct between the three groups in the absence of mobility changes, and in this case the normal animals initially follow the same trajectory as the SNI animals and then switch over to mimic the preference of Carrageenan animals. On both tasks, at this relatively mild challenge level, the Carrageenan animals avoid the challenge most and shift their preference away from the dark; while the SNI animals exhibit the least sensitivity to the challenge and continue to spend more time in the dark. Given that the behavioral outcome in this case depends on a contrast between two independent dimensions (light vs. heat in one case; and light vs. inclination in the other), differences along both dimensions need to be considered as a possible explanation for the results. On the ThermalTrack for 35°C challenge, one possible explanation of the outcome is that SNI animals are more averse to the light chamber (a change in the affective salience of the light/dark environment as a result of SNI injury), alternatively they are less sensitive, even compared to controls, to the heat, i.e. they actually prefer the warmer surface. Behavior on the 25°C ThermalTrack challenge resolves between these options. Given that in this challenge SNI animals spend more time in the light than Carrageenan or control animals, implies that if there is a shift in light/dark preference then it is in the opposite direction than observed for the 35°C challenge. Therefore, the simplest explanation for the outcome in 35°C challenge is that the SNI animals prefer the warmer surface or avoid the cooler surface. With the same logic we can state that the Carrageenan animals prefer the cooler surface at the cost of staying in the brighter chamber. With a similar logic we can conclude that the SNI animals are not bothered with the 25° inclination and continue to remain mainly in the dark chamber; while this challenge significantly affects the Carrageenan animals who switch their preference away from the dark chamber. Note the large increase in variability on both tests and for time in dark and for mobility, when animals are tested on these mild challenges (35°C or 25° inclination) in contrast to the corresponding neutral challenges, which is likely a consequence of the difficulty of making a decision within an ambivalent environment.

At the highest thermal challenge studied, 42°C, the behavior for time spent in the dark is very similar to the 35°C challenge, in that the SNI group still shows relatively more preference towards the darker (cooler) chamber in contrast to the Carrageenan group, and the control group has an intermediate behavior. In this case the SNI animals also show higher rate of switching events, adding further evidence for the notion that SNI animals are less affected by the thermal challenge. At the highest inclination challenge studied 55°, we observe a large interaction effect between time and groups, showing that SNI and Carrageenan animals stop climbing the high incline after initial attempts, while the normal animals still spend some time in the highly inclined dark chamber. Therefore, at this incline challenge both injured groups show decreased ability on the motoric challenge. On the ThermalTrack at temperatures intermediate between 35°C and 42°C, the time spent in the dark has the same pattern as at 35°C or 42°C, although the differences between SNI and Carrageenan animals are not significant. In contrast, performance on the AngleTrack at intermediate challenge levels remains hard to interpret.

Given these inter-relationships between time evolution of chamber preference and shifts in mobility, we attempted to derive a unifying metric that summarizes overall behavior. Normalized path length incorporates time evolution in preference and in mobility into an integrated single scale, which could be thought of as calculating the overall energy used for any given challenge on each tests. With this scale we can directly compare performance on the two tests and observe differences between them: on the ThermalTrack normalized path length is low for low challenge levels and increases at higher challenge levels; an opposite pattern is seen for AngleTrack. Moreover, on the ThermalTrack normalized path length changes in a continuous fashion from normal to SNI to Carrageenan animals.

### Behavioral implications of the results

The behavior modulation by thermal and motor challenges reveals interesting new properties regarding SNI and Carrageenan groups. The SNI group exhibits an overall affinity to innocuous heat since these animals spend relatively more time on the heated surface than normal animals. This result is very similar to recent observations in rats exposed to bilateral neuropathic injury, chronic constriction injury (CCI), which exhibit a relative aversion to cold in a 2-compartment shuttle box with one floor heated to 45°C and the other floor cooled to 10°C [10], implying that both SNI and CCI animals show hyperalgesia to cold. In contrast Carrageenan animals spend less time on the heated surface than normal animals, implying that they are hyperalgesic to heat. Thus, heat seems to be relatively favored by SNI and CCI animals and avoided by Carrageenan animals.

The motoric challenge affects the two pain animal groups similarly as on the heat challenge, although the differences are less prominent: SNI animals' behavior is at or slightly above the normal animals' preference of the challenge inclinations; while Carrageenan animals generally spend less time than the other two groups in the inclined chamber. This is a rather surprising outcome since part of our initial hypothesis was that animals with nerve injury that has differentially affected motor branches, i.e. the SNI group, should exhibit a larger deficit on the motoric challenge. Instead we observe the opposite, i.e. the inflammatory injury group showing more deficit than the neuropathic. It is possible that the contralateral intact limb adequately compensates the motor deficit, on this relatively simple motoric task. However, at least for the 25° inclination, given that SNI animals prefer the inclined surface even in comparison to controls, suggests dulling of sensitivity of SNI animals to the motoric challenge. In future studies it would be informative to explore performance on the motoric challenge when the SNI injury is induced bilaterally, eliminating contralateral compensation, as was recently reported for a different operant pain task [10], and alternatively to contrast SNI animals' behavior on this task to animals with a minimal motor injury but still exhibiting neuropathic pain-like behavior [11].

We did not find any reliable relationship between touch sensitivity and behavior on either ThermalTrack or AngleTrack. This may partially be due to technical issues, like variability of touch thresholds determined by Von Frey test. On the other hand distinct impact of each of the operant tasks on the animals' preference suggests that we are tapping into mechanisms more complex than spinal cord reflexes, and it would be surprising if limb withdrawal responses could be directly correlated to these complex behaviors. There is now emerging evidence from multiple groups indicating that pain as assessed on operant tasks can be distinct from more classical outcome measures like local tactile or thermal thresholds which are undoubtedly based on reflexive responses [10,12-15].

### Similarities of the two tasks to other pain assessing devices

ThermalTrack and AlgoTrack are variants on other operant pain assessing tasks introduced recently [1,16-20], all of which assess the impact of the painful state on the organism in general and thus take into consideration cortical circuitry. The ThermalTrack shares many similarities with an earlier thermal operant task [17], since both devices assess cost of heat on place preference. However, Mauderli et al. [17] have explored mainly effects of noxious heat or cold, while here we look at the effects of innocuous heat on place preference. Moreover, the earlier device incorporates complex behavioral signs of pain, guarding and licking [17,21], while here we sacrifice such measures to measure behavior automatically.

Cost of pain on motor behavior is traditionally measured by a simple motor performance, like Rotarod [22]. Such devices test the motor capability of animals in pain but not the modulation of place preference by motor challenges. To our knowledge AngleTrack is the first demonstration of the impact of a motor challenge on the natural preference of rats. From common everyday experience the expectation was that animals in pain should exhibit reduced preference to the dark, which requires climbing the inclined surface. We do observe this in the Carrageenan animals but, surprisingly, not or minimally in the SNI animals.

Here we have sought to eliminate the need for initial training as required for our earlier task [1], and in another variant of the task [18]. This makes studying large numbers of animals more straightforward. Moreover, since the only task the experimenter performs is placing the animal in the operant box, he/she cannot influence the outcome measures.

In summary, we present two fully-automated operant tasks, one probing the impact of heat on place preference, and the other the impact of a motoric challenge on place preference. The results suggest that neuropathic injury pain is minimally affected by the motor challenge and shows preference to innocuous heat, while inflammatory injury pain shows aversion to innocuous heat and to motoric challenge. These observations imply specific mechanistic differences between the two types of pain, but require further confirmatory studies, using analgesic manipulations, as well as testing other rodent pain models. Moreover, the operant task and related outcome measures can be readily extended to examine the impact of other dimensions on place preference, such as coldness, wetness, or mechanical roughness, providing a much richer assessment of pain conditions on the organism.

## Methods

The basic technique introduced in this paper involves an operant conditioning task, i.e.: modifying an animal behavioral pattern by the application of positive and/or negative stimuli. Our paradigm takes advantage of rats' natural preference for dark environments. This preference is challenged in various degrees by altering the environment while monitoring the animal behavior.

The test consists of introducing the rat into a box (see detailed description below) divided into dark and bright compartments. The challenge in the motoric task is to force the animal to climb uphill to satisfy its natural preference by tilting the box at different angles (thus the task is termed "AngleTrack"). The thermal task also consists of two identical compartments, where the floor of the dark compartment is heated relative to the bright compartment (this task is called "ThermalTrack"). The animal's position is monitored by a set of infrared sensors and the information collected by an automated computer system for the 30-minute duration of the test. The test was repeated in each animal with different inclination angles and temperatures with the objective of determining which challenge levels best differentiate various pain states.

### AngleTrack apparatus

This apparatus is made of two attached standard rat laboratory cages. Each compartment is a plastic box, 22 cm. wide, 22 cm. high, and 42 cm. long, covered by a perforated lid. One of the boxes is painted black to create the dark compartment, while the other is left transparent to create the bright compartment. In order to prevent the animal sliding at high angles, the bottom of the box is ridged to enhance traction. A wooden post was used to support the raised (dark) end of the Track. Both compartments are equipped with infrared sensors to detect entry of the animal. The sensors are connected to a data acquisition device that logs the animal's entry times throughout the experiment (1 Hz sampling rate).

### ThermalTrack apparatus

This apparatus is a 15 cm wide by 15 cm high by 48 cm long box, with clear plastic sides and cover, half the length is painted black, the other half left transparent. The bottom of the box consists of two aluminum plates, 15 cm wide by 24 cm long, each with a heating element glued to the underside and a thermocouple embedded within. The temperature of each plate is controlled independently by a PID controller (ODGEN ETR-3400, Arlington Height, Illinois). Similar to the AngleTrack, both compartments are equipped with infrared detectors connected to a data acquisition device to log the position of the animal (sampling rate 1 Hz.) during the experiment.

### Animals and pain models

Adult male Sprague Dawley rats (420–450 g, Harlan, Indianapolis, Indiana) were used, three groups of 12 animals each: 1) Healthy, 2) Neuropathic pain, and 3) Inflammatory pain. The first group of healthy rats received no treatment. In the second group of animals, neuropathic pain was induced in the left hind paw using the Spared Nerve Injury model (SNI) by severing the tibial nerve branch and the common peroneal nerve branch of the left sciatic nerve [23]. SNI surgery was performed one month before the beginning of the experiment, allowing enough time for development and stabilization of the neuropathic manifestations. In the third group of animals, inflammatory pain was induced in the left hind paw using an intraplantar injection of Carrageenan solution (100 μg of Carrageenan in 10 μl of saline per injection). Injections were performed 12 hours before the first day of the experiment to allow for the development of the inflammatory condition.

### AngleTrack experiment

Five different inclination angles were used in these experiments: 0°, 25°, 35°, 45°, and 55°. All rats were tested, for a period of 30 minutes, once per day, once at each inclination setting, in quasi-random order to avoid learning behavior. Rats were always introduced into the bright compartment.

### ThermalTrack experiment

Five different temperature settings were used in these experiments. For all settings, the floor of the illuminated compartment was maintained at 25°C, while the floor temperature of the dark compartment was set to either 25, 35, 38, 40 or 42°C. All animals were tested, for a period of 30 minutes, once for every temperature setting, in quasi-random order, and placed in the bright compartment at the start of each experiment.

### Von Frey test

Mechanical sensitivity of the hind paw was measured in all animal groups by determining withdrawal thresholds to Von Frey filaments. A set of 18 filaments (Stoelting, Chicago, IL.), marked from 1.65 to 6.5, was used. The respective bending forces were in the range of 0.005 to 125.892 g. The animals were placed individually in a small (35 × 20 × 15 cm) plastic cage with an open wire mesh bottom. Before testing, the rats were left in the test cages for 15–20 min so that their grooming and exploratory behaviors cease and all four paws were placed on the ground. All tests were performed on the right (control) and left (ligated) hind paws. Von Frey filaments were applied perpendicularly to the plantar surface of the paw with an upward force just sufficient to bend the microfilament. Special care was taken to stimulate the lateral plantar surface, which is the area of the skin innervated by the sural nerve [23]. Paw withdrawals due to locomotion or weight shifting were not counted and such trials were repeated. The 50% threshold for each paw withdrawal was calculated as described by Chaplan et al. [24].

### Statistical analysis

The animals' position and switching between compartments are the main outcome parameters that were studied, as a function of time, level of motoric or thermal challenge, and type of peripheral injury. Figures 1, 2, 3 show the position, switches, and position and switches as a continuous time function generated from the behavior of all 12 animals for each group (control, SNI, Carrageenan), calculated as proportion of animals of each group, where each data point is derived from behavior within a 2-minute time-window (15 time-windows over 30 minutes of monitoring). These results are descriptive in nature.

To compare behavior statistically between different parameters we calculate the time spent in dark chamber and the number of switches between chambers for each animal in 5-minute time-windows, over 30 minutes of monitoring for each challenge level. We use 3-way repeated measures-multi-way analysis of variance (RM-MANOVA), where time windows are repeated measures, to examine the various factor effects and their interactions (Tables I, II, III, IV). To highlight the effect of challenge level on behavior, we present performance as time spent in the dark chamber and as the number of switches between chambers for each challenge level. This data is shown for each challenge level in figure 4, 5, 6, 7, with 95% confidence interval for each challenge level, group, and time-window. Statistically this translates to 2-way repeated-measures ANOVA for groups and time-windows. The outcome of this test is then used to calculate the statistical difference between the two types of injury (inflammatory, i.e. Carrageenan injection, vs. neuropathic, i.e. SNI injured) using a planned comparison post-hoc analysis.

Normalized path length is a more global measure derived from the animals' behavior. Path length is the distance traveled in a 2-dimensional space comprised of time spent in dark and number of switches between chambers (space shown in figure 3). The 2-dimensional space is normalized to 1 in each axis, then the path length is calculated by determining position in six 5-minute time-windows, and then adding the respective line lengths, which is the square root of the sum of the squares of difference in x and y positions, at 6 consecutive positions. Thus, the normalized path length integrates position and switching events to a single metric that can be viewed as the overall energy used by the animal for different challenges. Results of this analysis are shown in figure 8. We also examined the relationship between mechanical thresholds (Von Frey test) for the injured hind limb and outcomes on the operant tasks, using linear correlations.

## Competing interests

The author(s) declare that they have no competing interests.
